# Environmental, Dietary, Maternal, and Fetal Predictors of Bulky DNA Adducts in Cord Blood: A European Mother–Child Study (NewGeneris)

**DOI:** 10.1289/ehp.1408613

**Published:** 2015-01-27

**Authors:** Marie Pedersen, Michelle A. Mendez, Bernadette Schoket, Roger W. Godschalk, Ana Espinosa, Anette Landström, Cristina M. Villanueva, Domenico F. Merlo, Eleni Fthenou, Esther Gracia-Lavedan, Frederik-J. van Schooten, Gerard Hoek, Gunnar Brunborg, Helle M. Meltzer, Jan Alexander, Jeanette K. Nielsen, Jordi Sunyer, John Wright, Katalin Kovács, Kees de Hoogh, Kristine B. Gutzkow, Laura J. Hardie, Leda Chatzi, Lisbeth E. Knudsen, Lívia Anna, Matthias Ketzel, Margaretha Haugen, Maria Botsivali, Mark J. Nieuwenhuijsen, Marta Cirach, Mireille B. Toledano, Rachel B. Smith, Sarah Fleming, Silvia Agramunt, Soterios A. Kyrtopoulos, Viktória Lukács, Jos C. Kleinjans, Dan Segerbäck, Manolis Kogevinas

**Affiliations:** 1 Centre for Research in Environmental Epidemiology (CREAL), Barcelona, Spain; 2 Universitat Pompeu Fabra, Barcelona, Spain; 3 CIBER Epidemiología y Salud Pública (CIBERESP), Madrid, Spain; 4 INSERM (National Institute of Health and Medical Research), U823, Team of Environmental Epidemiology Applied to Reproduction and Respiratory Health, Institute Albert Bonniot, Grenoble, France; 5 Department of Nutrition, University of North Carolina at Chapel Hill, Chapel Hill, North Carolina, USA; 6 Department of Molecular Environmental Epidemiology, National Institute of Environmental Health, Budapest, Hungary; 7 Department of Toxicology, Maastricht University, Maastricht, the Netherlands; 8 IMIM (Hospital del Mar Research Institute), Barcelona, Spain; 9 Department of Biosciences and Nutrition, Karolinska Institute, Huddinge, Sweden; 10 Epidemiology, Biostatistics, and Clinical Trials, IRCCS AOU San Martino-IST-National Cancer Research Institute, Genova, Italy; 11 Department of Social Medicine, University of Crete, Heraklion, Greece; 12 Institute for Risk Assessment Sciences, Utrecht University, Utrecht, the Netherlands; 13 Department of Chemicals and Radiation,; 14 Department of Exposure and Risk Assessment, and; 15 Division of Environmental Medicine, Norwegian Institute of Public Health, Oslo, Norway; 16 Department of Public Health, University of Copenhagen, Copenhagen, Denmark; 17 Bradford Institute for Health Research, Bradford Royal Infirmary, Bradford, United Kingdom; 18 Department of Epidemiology and Biostatistics, Imperial College London, London, the United Kingdom; 19 Leeds Institute of Genetics, Health and Therapeutics, University of Leeds, Leeds, United Kingdom; 20 Department of Environmental Science, Aarhus University, Roskilde, Denmark; 21 Institute of Biology, Medicinal Chemistry and Biotechnology, National Hellenic Research Foundation, Athens, Greece; 22 MRC-PHE Centre for Environment and Health, Department of Epidemiology and Biostatistics, School of Public Health, Imperial College London, London, United Kingdom; 23 Department of Toxicogenomics, Maastricht University, Maastricht, the Netherlands

## Abstract

**Background:**

Bulky DNA adducts reflect genotoxic exposures, have been associated with lower birth weight, and may predict cancer risk.

**Objective:**

We selected factors known or hypothesized to affect *in utero* adduct formation and repair and examined their associations with adduct levels in neonates.

**Methods:**

Pregnant women from Greece, Spain, England, Denmark, and Norway were recruited in 2006–2010. Cord blood bulky DNA adduct levels were measured by the ^32^P-postlabeling technique (*n* = 511). Diet and maternal characteristics were assessed via questionnaires. Modeled exposures to air pollutants and drinking-water disinfection by-products, mainly trihalomethanes (THMs), were available for a large proportion of the study population.

**Results:**

Greek and Spanish neonates had higher adduct levels than the northern European neonates [median, 12.1 (*n* = 179) vs. 6.8 (*n* = 332) adducts per 10^8^ nucleotides, *p* < 0.001]. Residence in southern European countries, higher maternal body mass index, delivery by cesarean section, male infant sex, low maternal intake of fruits rich in vitamin C, high intake of dairy products, and low adherence to healthy diet score were statistically significantly associated with higher adduct levels in adjusted models. Exposure to fine particulate matter and nitrogen dioxide was associated with significantly higher adducts in the Danish subsample only. Overall, the pooled results for THMs in water show no evidence of association with adduct levels; however, there are country-specific differences in results with a suggestion of an association in England.

**Conclusion:**

These findings suggest that a combination of factors, including unknown country-specific factors, influence the bulky DNA adduct levels in neonates.

**Citation:**

Pedersen M, Mendez MA, Schoket B, Godschalk RW, Espinosa A, Landström A, Villanueva CM, Merlo DF, Fthenou E, Gracia-Lavedan E, van Schooten FJ, Hoek G, Brunborg G, Meltzer HM, Alexander J, Nielsen JK, Sunyer J, Wright J, Kovács K, de Hoogh K, Gutzkow KB, Hardie LJ, Chatzi L, Knudsen LE, Anna L, Ketzel M, Haugen M, Botsivali M, Nieuwenhuijsen MJ, Cirach M, Toledano MB, Smith RB, Fleming S, Agramunt S, Kyrtopoulos SA, Lukács V, Kleinjans JC, Segerbäck D, Kogevinas M. 2015. Environmental, dietary, maternal, and fetal predictors of bulky DNA adducts in cord blood: a European mother–child study (NewGeneris). Environ Health Perspect 123:374–380; http://dx.doi.org/10.1289/ehp.1408613

## Introduction

Bulky DNA adducts are widely accepted as a sensitive biomarker of the biologically effective dose of exposure to genotoxic aromatic compounds, including polycyclic aromatic hydrocarbons (PAHs) and heterocyclic amines, from complex environmental exposures, including those in air, tobacco smoke, and diet (de Kok et al. 2002), and may be predictive of cancer risk in adults (Veglia et al. 2008). They provide an overall measure of exposure, absorption, and metabolic activation of a mixture of DNA adduct–forming compounds, integrated with repair of DNA damage of an individual (Farmer 1994; Phillips and Arlt 2007).

Bulky adducts have been detected in DNA from pregnant women (Godschalk et al. 2005; Pedersen et al. 2009, 2012a, 2013b; Topinka et al. 2009), placenta (Karttunen et al. 2010; Topinka et al. 2009), and cord blood (Godschalk et al. 2005; Hansen et al. 1993; Kovács et al. 2011; Pedersen et al. 2009, 2013b; Perera et al. 2011; Topinka et al. 2009). However, most of these studies are limited in size, and very little is known so far about modifiable predictors of the *in utero* formation and repair of these DNA adducts.

Maternal smoking (Godschalk and Kleinjans 2008; Hansen et al. 1993; Pedersen et al. 2009), exposure to traffic-related air pollution (Pedersen et al. 2009), and intake of meat with a blackened surface (Pedersen et al. 2012a) have been associated with higher levels of bulky DNA adducts in newborns. Diet is a significant source of exposure to agents that may modulate adduct formation toward either increase or decrease. Possible sources of these adducts are PAHs and other bulky DNA adduct–forming compounds that can be produced during cooking of certain foods such as meats and fish, and they also occur commonly as environmental contaminants especially of leafy plants, cereals, and shellfish [European Food Safety Authority (EFSA) 2008]. Another possible source of these adducts is related to oxidative processes (Arif et al. 2006), which may be modulated by dietary antioxidants.

We previously reported that higher levels of bulky DNA adducts in cord blood were significantly associated with a reduction in birth weight (Pedersen et al. 2013b). Therefore, to be able to prevent exposures and conditions underlying the DNA adduct formation *in utero* and subsequent health effects in offspring, more knowledge is needed on the main sources of DNA-damaging and -modifying factors. Given that a broad range of factors may affect the levels of bulky DNA adducts in neonates, we selected various maternal, fetal, dietary, and environmental (i.e., air and water pollution) factors known or hypothesized to affect *in utero* adduct formation and repair. In the present study, we investigated the relationships between these potential predictors and DNA adduct levels in white blood cells from neonates to explore and identify those preventable factors, which may be of importance for the bulky DNA adduct levels. We included exposures to compounds such as trihalomethanes (THMs) drinking-water disinfection by-products, which may not all form adducts themselves, but could modify the level of phase 1 and 2 enzymes (Allis et al. 2002; Richardson et al. 2007) and hereby interact with the metabolism of other compounds leading to a change in total adduct levels.

## Methods

*Study population*. The study was conducted as a part of the NewGeneris project that explored the impact of diet during pregnancy on child health (Merlo et al. 2009). Pregnant women were enrolled in the period from 2006 through 2010 at 11 maternity units located in Heraklion, Greece; Barcelona and Sabadell, Spain; Bradford, England; Copenhagen, Denmark; and Oslo, Norway (Pedersen et al. 2012b).

Detailed information on maternal characteristics and diet was obtained from questionnaires collected before or around the time of delivery (Table 1). Information on birth weight, gestational age, infant sex, and mode of delivery was obtained from birth records.

**Table 1:** Study population characteristics (*n* = 511) and DNA adduct levels (*n*/10^8^ nucleotides).

Predictor	*n*^*a*^ (%)	Median	Minimum–maximum	*p*-Value^*b*^
Country				< 0.001
Greece	54 (10.6)	12.5	0.8–43.9
Spain	125 (24.5)	11.6	1.1–87.5
Norway	58 (11.4)	5.5	1.2–22.3
England	79 (15.5)	8.8	0.6–52.7
Denmark	195 (38.2)	6.4	0.8–42.7
Maternal ethnicity (*n *= 508)				0.006
White	420 (82.7)	7.5	0.6–87.5
Nonwhite	88 (17.3)	9.3	1.0–78.4
Maternal age (years)				0.31
< 25	33 (6.4)	9.1	1.1–44.9
25–34	349 (68.2)	8	0.6–87.5
≥ 35	129 (25.2)	7.2	0.6–78.4
BMI (kg/m^2^) (*n *= 460)				0.003
Underweight (< 18.5)	19 (4.1)	8.4	0.8–35.5
Normal (> 18.5–25)	297 (64.6)	7	1.1–87.5
Overweight (> 25–30)	92 (20.0)	8.8	2.4–52.7
Obese (> 30)	52 (11.3)	11.2	0.6–44.9
Parity (*n *= 501)				0.008
0	180 (35.9)	7.1	0.8–87.5
≥ 1	321 (64.1)	8.3	0.6–78.4
Maternal education (*n *= 431)				0.43
Low	84 (19.5)	7.9	0.8–52.7
Middle	153 (35.4)	7.7	1.1–44.9
High	194 (45.1)	7.1	0.6–78.4
Maternal smoking during pregnancy (*n *= 505)				0.10
No	424 (84.0)	7.6	0.6–87.5
Yes	81 (16.0)	10.0	0.6–69.6
Maternal exposure to secondhand smoke (*n *= 488)^*c*^				0.03
No	309 (63.3)	7.3	0.6–87.5
Yes	179 (36.7)	8.8	0.6–60.5
Meat doneness (*n *= 466)^*c*^				0.08
Normal	291 (62.5)	7.1	0.6–52.7
Well-done	175 (37.5)	8.3	1.1–78.4
Tea (*n *= 481)^*c*^				< 0.001
None	152 (31.6)	10.8	0.8–87.5
Some	329 (68.4)	7.2	0.6–52.7
Dietary supplements (*n *= 466)^*c*^				0.10
Some	410 (88.0)	7.5	0.6–87.5
None	56 (12.0)	9.5	1.6–44.9
Season of delivery				< 0.001
March–May	127 (24.9)	7.1	0.6–24.4
June–August	85 (16.6)	11.7	1.3–87.5
September–November	202 (39.5)	7.0	0.8–78.4
December–February	97 (19.0)	9.4	0.6–43.7
Gestational age (weeks, *n *= 509)				0.50
≥ 37	493 (96.9)	7.8	0.6–87.5
< 37	16 (3.1)	8.6	1.3–43.7
Birth weight (g) (*n *= 510)				0.17
≥ 2,500	504 (98.8)	7.8	0.6–87.5
< 2,500	6 (1.2)	13.3	3.7–39.5
Mode of delivery (*n *= 509)				0.001
Vaginal	282 (55.4)	7.2	0.8–87.5
Cesarean section	227 (44.6)	8.4	0.6–60.5
Sex (*n *= 510)				0.025
Boy	272 (53.3)	8.7	0.6–87.5
Girl	238 (46.7)	7.3	0.8–78.4
^***a***^Total in specific variables may be < 511 because of missing values. ^***b***^*p*-Value from Kruskal–Wallis test for comparison across characteristic. ^***c***^All kinds.				

Cord blood DNA adduct measurements were available from 630 newborns born to women with singleton deliveries. From those we excluded 119 newborns for this analysis, because no information on maternal dietary habits was available or some mothers had a total energy estimate of < 500 or > 6,000 kcal/day.

Ethical approval was obtained from the ethics committee in each country. Written informed consent was obtained from all participating women.

*Dietary assessment*. In each country, the individual responses on the intake frequency of each food item during pregnancy were quantified as grams per day based on recipes and standard portion sizes (Pedersen et al. 2012b). Related food items were aggregated into 14 food groups (see Supplemental Material, Table S1). Subgroups of food items known to be sources of either higher levels of adduct-forming exposures (e.g., processed meat, meats, and leafy green vegetables), or higher levels of potential protective components (e.g., fruits rich in vitamin C, as well as their parent food groups), were evaluated separately and as a dietary score. Meat doneness was evaluated by use of photos and questions (Pedersen et al. 2012a).

The dietary score was created. For beneficial components (i.e., fruits, vegetables, and fish), women whose consumption was below the country-specific median were assigned a value of 0, and women whose consumption was at or above the median were assigned a value of 1. For components presumed to be high in adduct-forming exposures (i.e., meat, dairy products, cakes, cereals, and bread), each woman whose consumption was below the median received a value of 1, whereas each woman whose consumption was at or above the median was assigned a value of 0. Scores were added for each woman and ranged from 0 (lowest) to 7 (highest). The score was categorized as ≤ 2, low; 3–4, medium; or 5–7, high, healthy quality.

*Estimation of exposure to ambient air pollution and drinking-water disinfection by-products*. Fine particulate matter (PM ≤ 2.5 μm; PM_2.5_) and nitrogen dioxide (NO_2_) at the maternal home addresses during pregnancy were estimated with land-use regression (LUR) modeling developed in Greece, Spain, and England as part of the ESCAPE (European Study of Cohorts for Air Pollution Effects) project (Beelen et al. 2013; Eeftens et al. 2012; Pedersen et al. 2013a). Concentrations of PM and nitrogen oxides (NO_x_) in outdoor air were measured in Greece (2009–2010), Spain (2005–2006), and England (2007–2010). These air measurements were used together with geographic information system (GIS) variables on traffic characteristics, land use, population density, topography, and data from routine monitoring stations to model the exposure to ambient air pollution during the exact pregnancy periods in Spain and England. In the Greek subsample with bulky DNA adducts, there was too few routine monitoring data available to accurately back-extrapolate the LUR estimates, and the non–back-extrapolated LUR estimates were used assuming that the temporal variation between the pregnancy periods (November 2006–March 2008) and the air sampling periods (February 2009–February 2010)was minimal.

In Denmark, a dispersion model was used to estimate the air pollution as the sum of local air pollution from street traffic, urban background, and regional background taking into account meteorological factors using the human exposure modeling system AirGIS (Ketzel et al. 2011).

Area-level exposure to DBPs, mainly THMs, during pregnancy was estimated in Greece, Spain, and England as part of the HiWate (Health Impacts of long-term exposure to disinfection byproducts in drinking WATEr) project (Smith et al. 2009; Stayner et al. 2014; Villanueva et al. 2011). Tap-water samples from representative homes were collected repeatedly during four different time points between 2007 and 2009 in Heraklion, 2004 and 2006 in Sabadell, and 2004 and 2009 in Barcelona, and analyzed for total THMs, chloroform (CHCL_3_), bromoform (CHBr_3_), bromodichloromethane (CHBrCl_2_), and dibromochloromethane (CHBr_2_Cl) by gas chromatography/mass spectrometry.

Routine monitoring data from 2006 through 2011 were collected from Bradford, England. Average area-level (residential) exposure was modeled for each pregnancy together with trimester-specific exposure estimates (micrograms per liter) based on mother’s residence at birth and her exact pregnancy period. Information on maternal water use habits during pregnancy was combined with estimated residential water concentrations for the Greek participants to estimate maternal THM uptake integrated across all routes, including ingestion, dermal absorption, and inhalation (micrograms per day) (Stayner et al. 2014). Individual uptake could not be estimated for the participants from Spain because information on water habits was missing. For the participants from England, this integrated exposure metric accounted for boiling and filtering of the drinking water and incorporated THM uptake factors from biomonitoring studies for ingestion, showering, bathing, and swimming (Smith et al. 2009).

We were unable to estimate air and THM exposure for all participants in each area because data were missing on home addresses during pregnancy. Exposure to air pollution could not be estimated for participants from Norway because air measurements were missing. Furthermore, estimation of THM exposure in Denmark and Norway was not possible because there were no water measurements available in these study areas.

*Blood collection and bulky DNA adduct analysis (^32^P-postlabeling)*. Cord blood was collected immediately after delivery. DNA was isolated centrally and the levels of bulky DNA adducts were determined by using the ^32^P-postlabeling method with the nuclease P1 adduct enrichment version according to standardized protocols (Godschalk et al. 2005; Karttunen et al. 2010; Kovács et al. 2011). The protocols were harmonized and adjusted in an interlaboratory comparison study among the three ^32^P-postlabeling investigator laboratories (Pedersen et al. 2013b) and included the use of external benzo[*a*]pyrene-7,8-diol-9,10-epoxide (BPDE)–DNA standard [111 adducts in 10^8^ normal nucleotides (nt)]. All samples from Greece, Spain, and Norway and the Danish samples collected in 2006–2007 were analyzed at the National Institute of Environmental Health, Budapest, Hungary (61% of the samples); the Danish samples from 2009 were analyzed at the Karolinska Institute (21%); and the samples from England were analyzed at Maastricht University (18%). The individual level of DNA adducts was obtained as the average of at least two independent measurements. The detection limit of the assay was 0.1–0.3 adducts per 10^8^ unmodified nucleotides (*n*/10^8^ nt).

*Statistical analysis*. Descriptive statistics and histograms indicated that bulky DNA adduct levels in cord blood were not normally distributed, so the adduct levels were logarithmic-transformed. Linear regression models were used to evaluate the associations of the predictors with the adduct levels. The regression parameters estimated from the models were back-extrapolated using the exponential function and interpreted as ratios [mean ratio (MR)] of the mean DNA adducts in each level of the categorical variables relative to the reference group, whereas regression parameters estimated for continuous variables represent the proportional differences in DNA adduct levels associated with a 1-unit increase in continuous variables. Predictor variables were used as both continuous and categorical to assess potential nonlinear relationships with DNA adducts. In the adjusted models, potential confounders selected *a priori* were included: country, maternal smoking (no, yes), and prepregnancy body mass index (BMI) (kilograms per meter squared).

Main analyses were performed using the pooled data. In addition, for evaluation of air and THM exposure levels, we adjusted for season and performed country-specific analyses.

Stata S.E. version 12.1 was used for the statistical analyses (StataCorp, College Station, TX, USA), and we chose an alpha level of 5% to define statistical significance.

## Results

*Study population*. The study population was composed of neonates from Denmark (38%), Spain (24%), England (16%), Greece (11%), and Norway (11%). Mothers were predominantly white, multiparous, and nonsmoking, and few children were born before 37 weeks of gestation (*n* = 16) or with a birth weight < 2,500 g (*n* = 6) (Table 1).

*Maternal, fetal, and dietary factors in relation to adduct levels*. All cord blood samples (*n* = 511) had detectable levels of bulky DNA adducts, ranging from 0.6 to 87.5 adducts/10^8^ nt. Adduct levels were higher in neonates from southern Europe (i.e., Greece and Spain) than from northern Europe (i.e., England, Denmark, and Norway) (*p* < 0.001, Table 1). This difference between the samples from the south and north of Europe was also observed when study population was restricted to neonates of nonsmokers only [median (minimum–maximum): southern Europe: 13.0 (0.8–87.5) vs. northern Europe: 6.8 (0.6–52.7) adducts/10^8^ nt, *p* < 0.001]. Higher median adduct levels were observed in neonates whose mothers were nonwhite, obese or overweight, multiparous, and exposed to secondhand smoke (Table 1), and we observed a tendency for higher median adduct levels in neonates born to women who smoked compared with those of nonsmokers [10.0 (*n* = 81) vs. 7.6 (*n* = 424) adducts per 10^8^ nucleotide, *p* = 0.10]. Higher DNA adduct levels were also observed in newborns delivered by cesarean section versus vaginal birth; in boys versus girls; and in newborns born in June–August versus those born in other seasons (all *p* < 0.05).

For the full population in crude comparisons, adduct levels tended to be higher in the neonates of mothers who reported no intake of dietary supplements (*p* = 0.10) and a preference of well-done meat (*p* = 0.08). Higher adduct levels were observed in newborns of mothers who had higher intake of vegetable fats and processed meat, as well as all meat, whereas lower adduct levels were found in mothers who had higher intake of dried fruits, bread, and in children of mothers drinking tea (*p* < 0.05, Table 1; see also Supplemental Material, Table S1).

Most of the predictors identified in the crude comparisons were no longer significant after adjustment for country, maternal smoking, and BMI (see Supplemental Material, Table S2). However, country of birth, higher maternal BMI, delivery by cesarean section, male infant sex (Table 2), and low maternal intake of fruits rich in vitamin C and high intake of diary products as well as low adherence to healthy diet were associated with higher DNA adduct levels in adjusted models (Table 3).

**Table 2:** Maternal and fetal predictors in adjusted associations with bulky DNA adduct levels.

Predictor	*n*	MR^*a*^ (95% CI)	*p*-Value
Country
Greece	54	1.56 (1.22, 2.00)	< 0.001
Spain	105	1.61 (1.32, 1.95)	< 0.001
England	46	1.35 (1.04, 1.76)	0.02
Denmark	194	1.00 (reference)
Norway	56	0.82 (0.65, 1.03)	0.09
Maternal smoking
No	384	1.00 (reference)
Yes	71	0.91 (0.74, 1.13)	0.41
Maternal exposure to secondhand smoking
column1 indented	278	1.00 (reference)
column1 indented	157	1.02 (0.87, 1.20)	0.80
Prepregnancy BMI (per 1 kg/m^2^)	455	1.02 (1.00, 1.03)	0.02
Parity
0	172	1.00 (reference)
≥ 1	275	1.13 (0.98, 1.32)	0.10
Maternal education
Low	192	1.00 (reference)
Middle	147	0.94 (0.80, 1.11)	0.48
High	78	0.89 (0.72, 1.10)	0.28
Mode of delivery
Vaginal	260	1.00 (reference)
Cesarean section	193	1.34 (1.14, 1.59)	< 0.001
Season of delivery
March–May	110	1.00 (reference)
June–August	65	1.21 (0.92, 1.61)	0.17
September–November	189	0.88 (0.69, 1.12)	0.30
December–February	91	0.97 (0.75, 1.27)	0.84
Sex
Boy	237	1.00 (reference)
Girl	217	0.86 (0.74, 0.99)	0.03
Mean ratios (MRs) (95% CIs) for the categorical variables represent the proportional differences in bulky DNA adduct levels (*n*/10^8^ nucleotides) relative to the referent group. ^***a***^Adjusted for country, maternal smoking (no, yes), and prepregnancy BMI (kg/m^2^).			

**Table 3:** Dietary predictors in adjusted associations with bulky DNA adduct levels.

Predictor	*n*	MR^*a*^ (95% CI)	*p*-Value
Meat doneness
Normal	252	1.00 (reference)
Well-done	98	0.93 (0.77, 1.13)	0.47
Processed meat (per 1 g/day)
Low	149	1.00 (reference)
Middle	158	1.15 (0.97, 1.37)	0.11
High	137	1.15 (0.94, 1.40)	0.17
Dietary supplements
Some	392	1.00 (reference)
None	48	1.03 (0.78, 1.36)	0.82
Dairy products (per 1 g/day)	454	1.00 (1.00, 1.00)	0.06
Low	142	1.00 (reference)
Middle	153	1.10 (0.92, 1.32)	0.31
High	159	1.21 (1.00, 1.46)	0.05
Fruits with vitamin C (per 1 g/day)	453	1.00 (1.00, 1.00)	0.06
Low	147	1.00 (reference)
Middle	157	0.88 (0.74, 1.04)	0.13
High	149	0.83 (0.70, 0.99)	0.04
Healthy dietary score (0–7 no units)	455	0.93 (0.88, 0.98)	0.008
Low	106	1.00 (reference)
Middle	246	0.85 (0.71, 1.01)	0.06
High	103	0.78 (0.63, 0.96)	0.02
Mean ratios (MRs) (95% CIs) represent the proportional differences in bulky DNA adduct levels (*n*/10^8^ nucleotides) associated with a 1-unit increase in continuous variables, and for the categorical variables the MR and 95% CIs are relative to the referent group. ^***a***^Adjusted for country, maternal smoking (no, yes), and prepregnancy BMI (kg/m^2^).			

*Exposure to air pollution and trihalomethanes in relation to adduct levels*. Air pollution concentrations tended to be higher for participants from Spain than for those from Greece, England, and Denmark (see Supplemental Material, Table S3). Higher PM_2.5_ air pollution exposures were associated with higher adduct levels, but the association was only borderline significant (Table 4). Country-specific analysis resulted in significant associations in Denmark only (see Supplemental Material, Table S4).

**Table 4:** Environmental exposure concentrations and bulky DNA adduct levels in cord blood.

Pollutant (increment)	*n*	Median	Minimum–maximum	MR^*a*^ (95% CI)	*p-*Value
PM_2.5_ (per 5 μg/m^3^)	288	13.0	8.6–31.8	1.14 (0.99, 1.32)	0.07
NO_2_ (per 10 μg/m^3^)	291	20.1	8.2–103.3	1.01 (0.96, 1.07)	0.62
Area-level THMs (per 10 μg/L)	230	48.9	0.1–136.4	1.01 (0.98, 1.05)	0.51
Integrated uptake THMs (per 1 μg/day)	87	0.66	0.00–12.81	1.05 (0.39, 1.16)	0.30
Mean ratios (MRs) (95% CIs) represent the proportional differences in bulky DNA adduct levels (*n*/10^8^ nucleotides) associated with the indicated unit increase of mean exposure levels during the whole pregnancy at the home address. ^***a***^Adjusted for country, maternal smoking (no, yes), prepregnancy BMI (kg/m^2^), and season (spring, summer, autumn, and winter). Air pollution exposure is available for participants from Greece (*n *= 50), Spain (*n *= 99), England (*n *= 74), and Denmark (*n *= 65), but not for each and not for the participants from Norway. Area-level THM exposure is available for participants from Greece (*n *= 37), Spain (*n *= 114), and England (*n *= 79), but not for all and not for the participants from Denmark and Norway. The integrated uptake THM exposure is available from Greece (*n *= 38) and England (*n *= 49) only. Numbers are smaller in the regression models due to missing covariate data.					

THM concentrations in Heraklion were very low compared with those from Bradford, Sabadell, and Barcelona (see Supplemental Material, Table S3). It was not possible to estimate THM exposure in each study area because no data were available in Denmark and Norway. No significant associations were found for the pooled sample (Table 4). In England, higher area-level THM exposure during the third trimester was associated with higher adduct levels (see Supplemental Material, Table S4), but the association was of borderline significance (*p* = 0.10). No other associations were evident in models adjusted for season in addition to those model covariates selected *a priori*.

## Discussion

We explored potential predictors of bulky DNA adduct levels in white blood cells from cord blood in a large prospective multi-center European pregnancy study. Several potential modifiable factors, such as higher maternal BMI, high dairy product intake, low intake of healthy food and fruits rich in vitamin C, as well as delivery by cesarean section and country of birth, were associated with higher adduct levels. Heterogeneity across countries was evident for the associations with air pollution and THMs. Adjustment for country modified some associations, suggesting that multiple country-specific factors particularly for differences between southern and northern European populations, influence the adduct levels in newborns.

In our study population, the adduct levels tended to be higher in neonates born to women who smoked compared with those of nonsmokers, but the prevalence of smoking overall in the study was low. Although cigarette smoke is a major source of PAHs the presence of these adducts in cord blood is related not just to cigarette smoking of the mother. Statistically significant elevated levels of these or related DNA adducts in cord blood from newborns of mothers who smoked during pregnancy have been reported in some studies (Godschalk and Kleinjans 2008; Hansen et al. 1993; Pedersen et al. 2009), but not in others (Godschalk et al. 2005; Topinka et al. 2009). Significantly elevated levels of bulky DNA adducts have been detected in placentas from women who smoked compared with nonsmokers (Hansen et al. 1993; Topinka et al. 2009). Metabolic activation capacity of white blood cells may be a limiting factor for adduct formation when a saturation level is reached, resulting in leveling off of adduct formation as exposures increase (van Schooten et al. 1997). The lack of smoking-related increase in bulky DNA adduct levels might also be attributable to efficient repair in cord blood cells and to the fact that smoking is only a fraction of the total burden of potentially genotoxic substances (Daube et al. 1997). Food can also be an important source of PAHs (EFSA 2008). Furthermore, samples of smokers in these cord blood studies are small and limit the precision of results.

In our study, a statistically significant positive association was found between maternal BMI and DNA adduct levels. A previous study has evaluated this relationship, finding a similar pattern (Pedersen et al. 2009). BMI has been found to modulate the bulky DNA adduct levels in adult smokers (Godschalk et al. 2002). However, no statistically significant association between BMI and bulky DNA adduct levels was reported in a pooled study with 3,600 adults (Ricceri et al. 2010). Maternal BMI can be an independent risk factor or be a proxy of certain dietary/metabolic factors, which could influence *in utero* adduct formation.

Our finding that delivery by cesarean section was associated with higher adduct levels might be related to oxidative stress. Indeed, higher levels of total oxidant status (Saphier et al. 2013) and lipid hydroperoxide (Mutlu et al. 2011) have been found among newborns born by planned cesarean section. Although oxidative stress mainly leads to formation of non-bulky DNA adducts, bulky DNA adducts may also be produced (Arif et al. 2006; Berquist and Wilson 2012; Randerath et al. 1991).

A possible explanation for the fact that boys had higher levels of adducts than girls might be sex differences in metabolic enzyme activities (Liu et al. 2013). Boys may also be more vulnerable to maternal oxidative stress than girls, as suggested by a twin study (Minghetti et al. 2013).

Our finding that neonates from southern Europe had, on average, higher adduct levels than northern European neonates, is in line with findings in adults (Ricceri et al. 2010) and, as previously suggested, may reflect complex geographical differences in diet, food preparations and other factors such as exposure to ultraviolet light, and perhaps different genetic susceptibility toward environmental genotoxic agents.

Maternal intake of fruits rich in vitamin C was associated with lower adduct levels in cord blood, which is in line with previous findings; inverse associations with vitamin C have been reported in adults (Palli et al. 2000; Ragin et al. 2010), and higher intake of fruits and vegetables have been associated with lower bulky DNA adduct levels in adults (Palli et al. 2000; Peluso et al. 2008). Lower capacity to form DNA adducts has also been found in offspring of mice with a flavonoid-rich gestational diet (Vanhees et al. 2012). However, no associations were found with fruit intake (Pedersen et al. 2012b), and there were no associations observed between placental DNA adduct levels and vitamins A or C or β-carotene in plasma (Daube et al. 1997), but these two small studies did not estimate fruits rich in vitamin C. Likewise, the inverse association between dried fruits (rich in flavonoids) and adduct levels could suggest a potential inhibition of adduct formation by these nutrients.

We observed higher adduct levels among newborns of mothers with high intake of processed meats, a food group that is known to contain high levels of PAHs and heterocyclic amines [International Agency for Research on Cancer (IARC) 2010]. Supportive of this, we noted that adduct levels tended to be higher in neonates of women with preference for well-done meat (*p* = 0.08). Although higher cord blood adduct levels in relation to intake of meat with a blackened surface have been reported in one study (Pedersen et al. 2012b), a lack of any association with dietary PAH was also reported in another investigation by Perera et al. (2011). In addition to meats, dairy foods and leafy vegetables—which are potential sources of PAH exposure—have also previously been weakly associated with adduct levels (Falcó et al. 2003; Kazerouni et al. 2001).

We further evaluated the adherence to healthy food using a dietary score to take into account cumulative and interactive effects of beneficial and detrimental food patterns. We found an inverse association for high adherence to healthy food, and this approach may be particularly suitable in situations in which many dietary components may affect the outcome of investigation (Chatzi et al. 2012).

The lack of associations between maternal exposure to ambient air pollution and adduct levels in three of four countries was unexpected, because exposure to ambient air pollution has previously been associated with higher bulky DNA adduct levels in placenta (Topinka et al. 1997) and neonates (Pedersen et al. 2009). Significant associations were found in the Danish subsample for which the exposure assessment was based on a dispersion model. Although studies comparing dispersion and LUR models have typically found that LUR models perform at least as well as the dispersion models considered (Dijkema et al. 2011; Vardoulakis et al. 2003), it may be that more complete information on traffic and temporal variation related to meteorological factors has contributed to a better exposure assessment than detailed land use. All the same, ambient air is a complex and dynamic mixture that varies over space and time, and not all studies have reported elevated bulky DNA adduct levels in areas with poor air quality (Rossner et al. 2013). Finally, because of missing information, we were unable to estimate the exposure to air pollution for all participants, and our result is based on a subsample (*n* = 291 of 511), which could not include Norway.

THM exposure was available for a subset of the participants from Greece, Spain, and England only (*n* = 230 of 511), and not all of them had complete information on covariate data. We could not estimate THM exposure in Denmark and Norway. Overall, the pooled results for THMs in water show no evidence of association with adduct levels. There was a suggestion of an association with third-trimester exposure to area-level THMs in residential drinking water in England; however, this does not reach statistical significance, which may reflect small numbers in the analysis (*n* = 46). Findings were not consistent across countries, but this is not unexpected. THM levels in Greece were very low and contribute very little to exposure variability. It is quite possible that the study areas in England and Spain have different THM mixture profiles, and consequently different toxicity of exposures, which could explain lack of consistency between countries. In addition, only area-level THM exposure estimates were available for comparison between England and Spain. An exposure metric that takes into account individual water use would better reflect maternal THM exposure during pregnancy. However, in this study that metric was available for England only with reasonable exposure variability. There are no previous studies on THMs and bulky DNA adducts except for experimental evidence of mutagenicity (IARC 2004; Richardson et al. 2007). Another NewGeneris study from Crete has recently reported significant associations between THMs and micronuclei frequency in maternal lymphocytes, but the associations were not evident for the newborns (Stayner et al. 2014).

Potential limitations of our study could be related to the food frequency questionnaires, which rely on estimations of portion sizes and compositions of food. We acknowledge that interindividual differences in perception of meat doneness may have complicated our evaluation of meat doneness. Our assessment of exposure to air pollutants was limited to residential exposure to the most commonly measured pollutants, which served as a proxy for traffic and other combustion related emissions of exposures to DNA adduct–forming compounds. Exposures occurring elsewhere and during other time periods were not evaluated. Moreover, air pollution exposure was available only for 56% of the total study population, which did not include all participants from each study area or any participants from Norway. However, unadjusted and adjusted results were similar, and it is unlikely that there is any systematic bias, so we do not consider missing information on covariates a major concern for our study. Other potential limitations of our study were the inability to assess the intake of important dietary variables (i.e., vitamins E, A, and C) (Ragin et al. 2010). We had limited statistical power to evaluate the potential interactions with metabolic and repair genetic polymorphisms, which has been suggested to modulate the associations with air pollution (Topinka et al. 1997). We investigated a broad range of *a priori*–selected factors, and there might be issues related to multiple comparisons, and to the correlation between different factors and the extent to which they can act as surrogate for the exposures causing the effect. We think it is unlikely that the reported associations are chance findings, but we cannot rule out this possibility.

Key strengths of the present study relate to the harmonized protocols of the methodology, and the fact that cord blood was collected and processed using a common protocol (Merlo et al. 2009) in multiple study centers. Detailed information on maternal characteristics, such as diet, was collected in a manner that allowed pooling of data from participants enrolled in five different countries (Pedersen et al. 2012b). Air pollution was assessed using standardized, fine-scale land use regressions (Beelen et al. 2013; Eeftens et al. 2012) and a validated dispersion model (Ketzel et al. 2011). In addition to the sample size, having participants from different countries allows the testing of hypotheses under different settings.

In conclusion, our findings suggest that a combination of maternal, fetal, dietary, environmental, and also unknown country-specific factors influence the *in utero* formation of bulky DNA adducts. Several potential modifiable factors (e.g., higher maternal pre-pregnancy BMI, low maternal intake of healthy foods, and low intake of fruits rich in vitamin C) were identified as potential risk factors. The modification of these factors through public health policies might decrease further risks for the newborns later in life.

## Supplemental Material

(344 KB) PDF
