# Erratum: “Environmental, Dietary, Maternal, and Fetal Predictors of Bulky DNA Adducts in Cord Blood: A European Mother–Child Study (NewGeneris)”

**DOI:** 10.1289/ehp.124-A12

**Published:** 2016-01-01

**Authors:** Marie Pedersen, Michelle A. Mendez, Bernadette Schoket, Roger W. Godschalk, Ana Espinosa, Anette Landström, Cristina M. Villanueva, Domenico F. Merlo, Eleni Fthenou, Esther Gracia-Lavedan, Frederik-J. van Schooten, Gerard Hoek, Gunnar Brunborg, Helle M. Meltzer, Jan Alexander, Jeanette K. Nielsen, Jordi Sunyer, John Wright, Katalin Kovács, Kees de Hoogh, Kristine B. Gutzkow, Laura J. Hardie, Leda Chatzi, Lisbeth E. Knudsen, Lívia Anna, Matthias Ketzel, Margaretha Haugen, Maria Botsivali, Mark J. Nieuwenhuijsen, Marta Cirach, Mireille B. Toledano, Rachel B. Smith, Sarah Fleming, Silvia Agramunt, Soterios A. Kyrtopoulos, Viktória Lukács, Jos C. Kleinjans, Dan Segerbäck, Manolis Kogevinas

Environ Health Perspect 123(4):374–380 (2015); http://dx.doi.org/10.1289/ehp.1408613

In the “Results” section of the abstract, “median, 12.1 (*n* = 179) vs. 6.8 (*n* = 332) adducts per 108 nucleotides, *p* < 0.001” should have read “median, 12.1 (*n* = 179) vs. 6.8 (*n* = 332) adducts per 10^8^ nucleotides, *p* < 0.001.”

The published article has been updated online. *EHP* regrets this error.

