# Supplementary material for: Environmental, Dietary, Maternal, and Fetal Predictors of Bulky DNA Adducts in Cord Blood: A European Mother–Child Study (NewGeneris)
Source: Environ Health Perspect. 2015 Jan 27;123(4):374–80. doi: 10.1289/ehp.1408613 (PMC4383575; doi:10.1289/ehp.1408613)
Supplement: (344 KB) PDF [file ehp.1408613.s001.508.pdf]

Environmental Health Perspectives doi:10.1289/ehp.1408613

Supplemental Material

Environmental, Dietary, Maternal, and Fetal Predictors of Bulky DNA Adducts in Cord Blood: A European Mother–Child Study (NewGeneris)

Marie Pedersen, Michelle A. Mendez, Bernadette Schoket, Roger W. Godschalk, Ana Espinosa, Anette Landström, Cristina M. Villanueva, Domenico F. Merlo, Eleni Fthenou, Esther Gracia-Lavedan, Frederik-J. van Schooten, Gerard Hoek, Gunnar Brunborg, Helle M. Meltzer, Jan Alexander, Jeanette K. Nielsen, Jordi Sunyer, John Wright, Katalin Kovács, Kees de Hoogh, Kristine B. Gutzkow, Laura J. Hardie, Leda Chatzi, Lisbeth E. Knudsen, Lívia Anna, Matthias Ketzel, Margaretha Haugen, Maria Botsivali, Mark J. Nieuwenhuijsen, Marta Cirach, Mireille B. Toledano, Rachel B. Smith, Sarah Fleming, Silvia Agramunt, Soterios A. Kyrtopoulos, Viktória Lukács, Jos C. Kleinjans, Dan Segerbäck, and Manolis Kogevinas

Table S1. Maternal dietary intake during pregnancy and bulky DNA adduct levels.

Variable	n[a]	Maternal dietary intake (g/day) Median (Min-Max)	Bulky DNA adducts (n/10^{8}nt) Median (Min-Max)	p[b]
Fruits with vitamin C[c]	509	122 (0-1810)	NA	0.30
Low	170	35 (0-74)	8.1 (0.6-87.5)	
Middle	172	122 (75-193)	7.7 (0.6-69.6)	
High	167	288 (194-1810)	7.8 (0.8-44.9)	
Dried fruits[d]	492	3 (0-162)	NA	0.01
Low	167	0 (0-1)	9.4 (0.8-52.7)	
Middle	164	4 (1-9)	7.8 (0.6-87.5)	
High	161	22 (9-161)	7.2 (1.1-69.6)	
All fruits[e]	509	472 (35-4099)	NA	0.25
Low	170	204 (35-349)	7.8 (0.6-52.7)	
Middle	170	473 (350-598)	7.6 (1.2-87.5)	
High	169	894 (599-4099)	8.9 (0.8-69.6)	
Green leafy vegetables[f]	508	24 (0-436)	NA	0.05
Low	170	8 (0-13)	7.2 (0.6-39.6)	
Middle	177	24 (13-41)	8.5 (0.9-87.5)	
High	161	63 (42-436)	7.9 (0.6-78.4)	
All vegetables[g]	510	347 (6-4995)	NA	0.12
Low	170	158 (6-249)	8.0 (0.6-87.5)	
Middle	170	347 (250-264)	9.2 (0.6-78.4)	
High	170	647 (464-4995)	7.0 (1.0-60.5)	
Vegetable fat[h]	505	6 (0-327)	NA	<0.001
Low	187	1 (0-3)	6.4 (1.1-42.7)	
Middle	150	7 (3-14)	8.6 (0.6-87.5)	
High	168	28 (14-327)	9.4 (0.6-52.1)	
Fish[i]	500	40 (0-382)	NA	0.59
Low	167	13 (0-24)	7.8 (0.8-52.7)	
Middle	167	40 (24-57)	8.1 (0.6-69.6)	
High	166	85 (57-382)	7.6 (0.8-87.5)	
Shellfish[j]	408	4 (0-129)	NA	0.02
Low	152	0 (0-0)	8.2 (0.8-78.4)	
Middle	137	5 (1-7)	6.8 (0.8-43.9)	
High	119	17 (7-129)	8.2 (1.1-87.5)	
Processed meat[k]	497	5 (0-214)	NA	0.03
Low	166	0 (0-2)	7.4 (0.8-39.6)	
Middle	166	5 (2-9)	7.7 (0.8-60.5)	
High	165	21 (9-214)	8.9 (0.6-87.5)	
All meat[l]	511	121 (0-738)	NA	0.05
Low	171	65 (0-93)	7.6 (0.6-52.7)	
Middle	170	121 (93-145)	7.4 (0.8-60.5)	
High	170	183 (145-738)	8.8 (0.6-87.5)	
Bread[m]	510	112 (0-517)	NA	0.01
Low	170	46 (0-73)	9.0 (0.6-78.4)	
Middle	171	112 (73-160)	8.4 (1.4-87.5)	
High	169	220 (163-517)	6.8 (0.8-41.3)	
Cereals[n]	508	102 (11-1258)	NA	0.22
Low	170	57 (11-82)	8.4 (0.6-78.4)	
Middle	169	102 (83-130)	8.1 (0.8-87.5)	
High	169	165 (131-1258)	7.2 (1.0-60.5)	

Variable	n[a]	Maternal dietary intake (g/day) Median (Min-Max)	Bulky DNA adducts ($n/10^8 nt$) Median (Min-Max)	p[b]
Cakes[o]	508	27 (0-598)	NA	0.70
Low	170	8 (0-17)	8.2 (0.6-87.5)	
Middle	160	28 (18-40)	7.7 (0.8-44.9)	
High	169	58 (41-598)	7.7 (0.6-60.5)	
Dairy products[p]	510	318 (0-2294)	NA	0.09
Low	170	83 (0-206)	7.3 (0.6-52.7)	
Middle	170	318 (206-517)	8.1 (0.6-69.6)	
High	170	653 (517-2294)	8.4 (0.9-87.5)	
Energy (kcal)	511	1267 (622-5918)	NA	0.64
Low	171	1712 (622-2095)	8.1 (0.6-78.4)	
Middle	170	2469 (2098-2796)	7.7 (0.6-87.5)	
High	170	3369 (2796-5918)	7.6 (1.0-43.9)	
Coffee (n=451)[q]				0.21
None	100	(22.2)	7.9 (1.1-43.9)	
Some	351	(77.8)	7.6 (0.6-87.5)	
Alcohol (n=509)[q]				0.51
None	373	(73.3)	7.9 (0.6-87.5)	
Some	136	(26.7)	7.7 (0.6-69.6)	
Healthy dietary score[r]				0.11
Low	112	(21.9)	8.3 (1.4-87.5)	
Middle	283	(55.4)	8.1 (0.6-69.6)	
High	116	(22.7)	6.9 (1.0-43.7)	

NA not applicable

[a]Total in specific variables may be less than 511 because of missing values. [b]P-value from Kruskall-Wallis test for comparison across tertiles derived from pooled sample. [c]Citrus fruits, berries, fresh and packed juice from citrus fruits. [d]Dates, apricots, raisins, figs and other dried and preserved fruits. [e]Sum of C-vitamin rich fruits, other fruits (banana, apple, pear, stone fruits, melon, fig, grapes, tropical fruits, fruit salad, fresh and packed fruit juice) and dried fruits. [f]Lettuce, spinach, endives, parsley, herbs, cabbage and mixed dishes. [g]Sum of green leafy vegetables, tomato, cucumber, carrots, beetroot, okra, leek, onion, garlic, pepper, cauliflower, broccoli, celery, Brussels sprouts, eggplant, zucchini, green beans, mushrooms, peas, corn, artichokes, avocado, asparagus, swede, and mixed dishes. Both raw and cooked dishes. Boiled, fried, roasted, salad and mixed dishes. [h]All kinds of added oils. [i]Tuna, cod, hake, sole, salmon, herring, plaice, halibut, perch, mackerel, sardine, squid, cuttlefish, octopus, and mixed dishes. Raw, cooked, canned and salted. [j]Clams, mussels, cockles, shrimps, crab and mixed dishes. Raw and cooked dishes. [k]Salami, bacon, ham, mortadella, sausages, smoked and cured meats. [l]Veal, beef, pork, goat, lamb, rabbit, hare, reindeer, elk, offal, tripe, brain, steak, fillet, roast, minced meat, pate, chicken, turkey, goose, hunt birds, game and mixed dishes. Hot and cold dishes including topping and main meals. [m]White bread, brown bread, crisp bread, crackers, chapattis, nan bread, pitta bread and mixed dishes. [n]Breakfast cereals, cornflakes, all bran,

muesli, pasta, rice, and other cereals. [o]Cakes, pastry, donuts, scones, croissant, waffles, cream crackers, sweet and chocolate biscuits, baklava, flapjacks, sweet crepes and cookies. [p]All kinds of cheese, butter, milk, yoghurt, crème fraîche and other dairy products. Both fresh, preserved and condensed milk. [q]All kinds. [r]Score of beneficial components (i.e. fruits, vegetables and fish) and components presumed to be high in adduct forming exposures (i.e. meat, dairy products, cakes, cereals and bread). The score was categorized (1) ≤2, low; (2) 3–4, medium; (3) 5–7, high healthy quality.

Table S2. Bulky DNA adduct levels in relations to maternal, fetal and dietary predictors.

Characteristic	n[a]	MR (95% CI)[b]	p
Ethnicity			
White	382	1.00 (Ref.)	
Non-white	71	0.92 (0.73, 1.17)	0.50
Maternal age (per 1 years)	455	0.99 (0.91, 1.07)	0.80
25-30	312	1.00 (Ref.)	
<25	22	0.85 (0.61, 1.20)	0.36
>35	121	0.99 (0.84, 1.16)	0.89
Pre-pregnancy BMI (per 1 kg/m^2)	19	1.33 (0.91, 1.96)	0.14
Underweight	294	1.00 (Ref.)	
Normal	90	0.92 (0.71, 1.19)	0.51
Overweight	52	0.69 (0.42, 1.11)	0.13
Obese	453	0.97 (0.93, 1.02)	0.30
Gestational age (per 1 week)	438	1.00 (Ref.)	
≥37 weeks	15	1.14 (0.76, 1.70)	0.52
<37 weeks	454	1.00 (1.00, 1.00)	0.17
Birth weight (per 1 g)			
≥2.500 g	454	1.00 (Ref.) 1.00	
>2.500 g	4	1.96 (0.92, 4.17)	0.08
Dried fruits (per 1 g/day)	436	1.00 (1.00, 1.00)	0.87
Low	132	1.00 (Ref.)	
Middle	150	0.90 (0.75, 1.09)	0.30
High	154	0.90 (0.73, 1.09)	0.28
All fruits (per 1 g/day)	453	1.00 (1.00, 1.00)	0.54
Low	143	1.00 (Ref.)	0.88
Middle	159	0.99 (0.83, 1.17)	0.36
High	151	0.92 (0.76, 1.10)	0.91
Green leafy vegetables (per 1 g/day)	452	1.00 (1.00, 1.00)	
Low	152	1.00 (Ref.)	
Middle	152	1.18 (0.99, 1.42)	0.07
High	148	1.09 (0.90, 1.31)	0.38
All vegetables (per 1 g/day)	454	1.00 (1.00, 1.00)	0.02
Low	144	1.00 (Ref.)	
Middle	151	0.93 (0.77, 1.12)	0.43
High	159	0.94 (0.77, 1.16)	0.58
Vegetable fat (per 1 g/day)	449	1.00 (0.99, 1.01)	0.94
Low	183	1.00 (Ref.)	
Middle	130	1.02 (0.80, 1.28)	0.90
High	136	0.95 (0.73, 1.24)	0.70
Fish (per 1 g/day)	445	1.00 (1.00, 1.00)	0.11
Low	139	1.00 (Ref.)	
Middle	153	1.11 (0.92, 1.34)	0.29
High	153	0.95 (0.78, 1.17)	0.65
Shell fish (per 1 g/day)	386	1.00 (0.99, 1.00)	0.17
Low	146	1.00 (Ref.)	
Middle	131	0.92 (0.75, 1.12)	0.41
High	109	0.96 (0.77, 1.19)	0.72
All meat (per 1 g/day)	455	1.00 (1.00, 1.00)	0.70
Low	151	1.00 (Ref.)	
Middle	153	0.96 (0.81, 1.14)	0.65
High	151	1.12 (0.93, 1.35)	0.22
Bread (per 1 g/day)	454	1.00 (1.00, 1.00)	0.53
Low	138	1.00 (Ref.)	
Middle	154	1.01 (0.84, 1.21)	0.94
High	162	1.03 (0.84, 1.26)	0.77

Characteristic	n[a]	MR (95% CI)[b]	p
Cereals (per 1 g/day)	452	1.00 (1.00, 1.00)	0.99
Low	149	1.00 (Ref.)	
Middle	156	1.17 (0.98, 1.39)	0.08
High	147	1.00 (0.84, 1.19)	0.99
Cakes (per 1 g/day)	452	1.00 (1.00, 1.00)	0.52
Low	143	1.00 (Ref.)	
Middle	156	1.08 (0.90, 1.30)	0.41
High	153	1.08 (0.90, 1.31)	0.40
Energy (kcal)	455	1.00 (1.00, 1.00)	0.67
Low	146	1.00 (Ref.)	
Middle	156	1.00 (0.83, 1.20)	0.99
High	153	1.00 (0.82, 1.20)	0.97
Tea			
None	142	1.00 (Ref.)	
Some	291	0.90 (0.74, 1.10)	0.32
Coffee			
None	90	1.00 (Ref.)	
Some	325	0.97 (0.80, 1.17)	0.73
Alcohol			
None	323	1.00 (Ref.)	
Some	130	1.15 (0.97, 1.35)	0.10

Mean ratios (MR) and their 95% confidence intervals (CIs) represent the proportional differences in bulky DNA adduct levels ($n/10^8$ nucleotides) associated with a 1-unit increase in continuous variables and for the categorical variables the MR and 95% CIs are relative to the referent group. [a]Total in specific variables may be less than 511 because of missing values. [b]Adjusted for country, maternal smoking (no, yes) and pre-pregnancy BMI (kg/m^2).

Table S3. Concentrations of air pollution and trihalomethanes by country.

Exposure	Heraklion, Greece n	Median (min-max)	Sabadell and Barcelona, Spain n	Median (min-max)	Bradford, England n	Median (min-max)	Copenhagen, Denmark n	Median (min-max)
$PM_{2.5}$ (μg/m^3)								
LUR mean	50	14.4 (13.0-17.9)	NA	NA	NA	NA	NA	NA
Entire pregnancy	NA	NA	99	18.0 (11.8-31.8)	74	10.5 (8.6-14.7)	65	11.0 (9.4-13.3)
1st trimester	NA	NA	99	17.6 (10.4-30.8)	74	12.5 (8.6-17.9)	65	12.3 (8.8-15.2)
2nd trimester	NA	NA	99	18.1 (11.6-34.7)	74	13.3 (8.0-18.2)	65	10.7 (8.3-13.7)
3rd trimester	NA	NA	99	15.7 (10.0-34.0)	74	12.2 (8.8-19.1)	65	10.3 (7.0-15.1)
NO_2 (μg/m^3)								
LUR mean	50	11.6 (8.2-20.5)	NA	NA	NA	NA	NA	NA
Entire pregnancy	NA	NA	99	50.4 (20.1-103.3)	77	17.2 (12.1-25.3)	65	19.9 (9.1-34.9)
1st trimester	NA	NA	99	51.6 (20.3-108.8)	77	16.3 (10.1-26.7)	65	19.6 (9.3-36.8)
2nd trimester	NA	NA	99	50.8 (20.2-106.8)	77	18.8 (10.3-34.9)	65	20.1 (8.4-32.5)
3rd trimester	NA	NA	99	48.6 (17.3-118.3)	77	15.2 (8.9-32.0)	65	18.3 (6.3-36.2)
Area-level THMs (μg/L)								
Entire pregnancy	37	0.72 (0.08-7.09)	114	91.5 (42.2-136.4)	79	45 (41-54)	NA	NA
1st trimester	37	0.36 (0.02-4.25)	114	89.1 (35.8-130.2)	79	48 (32-64)	NA	NA
2nd trimester	37	0.26 (0.02-10.17)	114	93.4 (34.6-135.2)	79	40 (32-65)	NA	NA
3rd trimester	37	1.19 (0.10-10.41)	114	95.5 (38.0-159.2)	79	44 (32-65)	NA	NA
Integrated uptake THMs (μg/day)[a]								
Entire pregnancy	38	0.02 (0.00-0.49)	NA	NA	49	1.54 (0.31-12.81)	NA	NA
1st trimester	38	0.01 (0.00-0.11)	NA	NA	49	1.58 (0.31-12.63)	NA	NA
2nd trimester	38	0.01 (0.00-0.53)	NA	NA	49	1.44 (0.36-12.66)	NA	NA
3rd trimester	38	0.02 (0.00-0.83)	NA	NA	49	1.48 (0.31-13.15)	NA	NA

Na: not available; NO_2: nitrogen dioxide; $PM_{2.5}$: particulate matter with an aerodynamic diameter <2.5μm; THMs: trihalomethanes.

[a]Uptake of THMs per day integrated across all exposure routes, i.e. ingestion, inhalation and dermal absorption.

Table S4. Air pollution, trihalomethanes and bulky DNA adduct levels.

Exposure	Heraklion, Greece n	MR (95%CI)	p	Sabadell & Barcelona, Spain n	MR (95%CI)	p	Bradford, England n	MR (95%CI)	p	Copenhagen, Denmark n	MR (95%CI)	p
$PM_{2.5}$ (per 5 μg/m^3)												
LUR mean	50	1.04 (0.38, 2.85)	0.94									
Entire pregnancy	NA	NA	NA	84	1.17 (0.87, 1.58)	0.29	43	1.13 (0.49, 2.64)	0.77	65	5.99 (1.72, 20.84)	0.01
1st trimester	NA	NA	NA	84	1.10 (0.84, 1.43)	0.48	43	1.56 (0.91, 2.68)	0.10	65	2.59 (1.21, 5.54)	0.02
2nd trimester	NA	NA	NA	84	1.22 (0.91, 1.63)	0.19	43	1.32 (0.79, 2.21)	0.29	65	1.66 (0.71, 3.89)	0.24
3rd trimester	NA	NA	NA	84	0.91 (0.70, 1.19)	0.48	43	1.13 (0.68, 1.87)	0.63	65	2.20 (0.85, 5.70)	0.10
NO_2 (per 10 μg/m^3)												
LUR mean	50	0.77 (0.43, 1.39)	0.38									
Entire pregnancy	NA	NA	NA	84	0.98 (0.86, 1.12)	0.79	45	0.85 (0.44, 1.64)	0.62	65	1.27 (0.93, 1.74)	0.13
1st trimester	NA	NA	NA	84	1.02 (0.90, 1.16)	0.77	45	1.50 (0.87, 2.58)	0.14	65	1.26 (0.95, 1.67)	0.11
2nd trimester	NA	NA	NA	84	0.96 (0.84, 1.09)	0.51	45	0.83 (0.50, 1.39)	0.48	65	1.20 (0.89, 1.63)	0.23
3rd trimester	NA	NA	NA	84	0.88 (0.79, 0.98)	0.02	45	0.88 (0.55, 1.40)	0.57	65	1.33 (0.98, 1.82)	0.07
Area-level THMs (per 10 μg/L)												
Entire pregnancy	37	1.06 (0.29, 3.86)	0.93	98	1.00 (0.92, 1.02)	0.99	46	0.70 (0.22, 2.22)	0.54	NA	NA	NA
1st trimester	37	0.64 (0.02, 21.29)	0.80	98	0.98 (0.89, 1.07)	0.60	46	0.80 (0.60, 1.07)	0.13	NA	NA	NA
2nd trimester	37	0.93 (0.30, 2.83)	0.89	98	0.99 (0.91, 1.09)	0.91	46	0.72 (0.39, 1.32)	0.28	NA	NA	NA
3rd trimester	37	1.09 (0.50, 2.38)	0.81	98	1.02 (0.95, 1.10)	0.56	46	1.28 (0.96, 1.71)	0.10	NA	NA	NA
Integrated uptake THMs[a] (per 1 μg/day)												
Entire pregnancy	37	0.08 (0.00, 1.63)	0.10	NA	NA	NA	45	1.03 (0.93, 1.13)	0.58	NA	NA	NA
1st trimester	37	0.00 (0.00, 17.59)	0.16	NA	NA	NA	45	1.02 (0.94, 1.12)	0.60	NA	NA	NA
2nd trimester	37	0.07 (0.00, 1.18)	0.05	NA	NA	NA	45	1.03 (0.94, 1.13)	0.58	NA	NA	NA
3rd trimester	37	0.25 (0.04, 1.50)	0.09	NA	NA	NA	45	1.03 (0.93, 1.13)	0.58	NA	NA	NA

Na: not available; NO_2: nitrogen dioxide; $PM_{2.5}$: particulate matter with an aerodynamic diameter <2.5μm; THMs: trihalomethanes.

Mean ratios (MR) and their 95% confidence intervals (CIs) represent the proportional differences in bulky DNA adduct levels (n/10^8nucleotides) associated with the indicated unit increase in mean exposure levels at the home address.

Adjusted for country, maternal smoking (no, yes), pre-pregnancy BMI (kg/m^2) and season (spring, summer, autumn and winter).

Numbers are smaller than in Table S3 due to missing covariate data.

[a]Uptake of THMs per day integrated across all exposure routes, i.e. ingestion, inhalation and dermal absorption.
